# Clinical relevance and translational impact of reduced penetrance in genetic movement disorders

**DOI:** 10.1515/medgen-2022-2128

**Published:** 2022-08-12

**Authors:** Sebastian Heinzel, Deborah Mascalzoni, Tobias Bäumer, Daniela Berg, Meike Kasten, Norbert Brüggemann

**Affiliations:** Department of Neurology, Christian-Albrechts University of Kiel, Kiel, Germany; Institute for Biomedicine, Eurac Research, Affiliated Institute of the University of Lübeck, Bolzano, Italy; Center for Research Ethics and Bioethics, Department of Public Health and Caring Sciences, Uppsala University, Uppsala, Sweden; Institute of Systems Motor Science, University of Lübeck, Lübeck, Germany; Department of Psychiatry and Psychotherapy, University of Lübeck, Lübeck, Germany; Institute of Neurogenetics, University of Lübeck, Lübeck, Germany; Department of Neurology, Center for Brain, Behavior and Metabolism, University of Lübeck, Lübeck, Germany

**Keywords:** penetrance, expressivity, genetic counseling, recall-by-genotype, translational

## Abstract

Reduced penetrance is an important but underreported aspect in monogenic diseases. It refers to the phenomenon that carriers of pathogenic variants do not manifest with an overt disease. Clinical expressivity, on the other hand, describes the degree to which certain disease characteristics are present. In this article, we discuss the implications of reduced penetrance on genetic testing and counseling, outline how penetrance can be estimated in rare diseases using large cohorts and review the ethical, legal and social implications of studying non-manifesting carriers of pathogenic mutations. We highlight the interplay between reduced penetrance and the prodromal phase of a neurodegenerative disorder through the example of monogenic Parkinson’s disease and discuss the therapeutic implications.

## Introduction

The treatment of patients with genetic diseases has changed fundamentally in recent years. Advances in diagnostics have led to the discovery of new disease-causing genes and thus disease-specific pathways, and several gene-specific therapies have already been introduced.

An important aspect in the application of translational therapies, which has not been considered very much so far, is the reduced penetrance of disease-causing mutations. Reduced penetrance refers to the phenomenon that carriers of pathogenic variants do not manifest with an overt disease. Clinical expressivity, on the other hand, describes the degree to which certain disease characteristics are present ranging from subclinical disease signs to atypical phenotypic presentations. Reduced penetrance therefore refers not only to the distinction between healthy and diseased, but also takes into account the intermediate stages from practically and clinically non-significant abnormalities to the full phenotypic expression. Another aspect is the time at which a monogenic disease manifests clinically. The age-dependent penetrance can vary widely, despite the presence of a mutation within the same gene. Genetic modifiers as well as environmental factors and gene-environment interactions are discussed as causes for reduced penetrance and altered expressivity.

Inherited degenerative diseases contribute to the complexity of our understanding of penetrance and expressivity. These diseases are usually diagnosed at a time when significant degenerative and irreversible damage has already occurred. In this prodromal phase which precedes the clinical diagnosis, nonspecific symptoms or abnormal biomarkers may be detectable, indicating the underlying disease process. Genetic testing allows the identification of mutation carriers but it often remains elusive whether a carrier is in or will enter the prodromal phase, and whether a carrier will develop an overt phenotype in the future. Treating non-manifesting carriers with disease-modifying therapies thus represents a particular challenge. On the one hand, there is the possibility to intervene earlier in the disease process and to slow down the progression of the degeneration, which could positively influence the course of the disease. On the other hand, only carriers who are very likely to enter clinical disease stages should be treated. The treatment of mutation carriers who will never develop the disease should in contrast be avoided due to potential side effects and unnecessary treatment costs.

In this article, we would like to address the clinical relevance and translational impact of reduced penetrance. Specifically, we discuss the implications of reduced penetrance on genetic testing and counseling and outline how reduced penetrance can be investigated in rare diseases using databases and large cohorts. In another section, we review the prodromal phase of monogenic Parkinson’s disease as a model for an inherited neurodegenerative disease and its relationship to reduced penetrance. Moreover, ethical and legal implications of studying non-manifesting carriers of pathogenic mutations are outlined and discussed.

## Genetic counseling

Reduced penetrance plays an important role in genetic counseling of family members of patients with monogenic diseases. If such a family member wishes to be tested for a disease-causing mutation for disease prediction purposes, the possibility of reduced penetrance and expressivity needs to be part of the counseling process. In this context, the higher the penetrance of a mutation, the more accurate the prediction of whether a carrier will develop the disease. An example for a highly penetrant genetic disorder is Huntington’s disease (HD), where every mutation carrier with more than 40 CAG repeats in the HTT gene will eventually develop the phenotype [1]. Due to the autosomal dominant inheritance there is in fact a 50 % likelihood of not only inheriting the mutation, but also of definitely developing the disease. The situation is different for genetic forms of dystonia. In the case of DYT1 dystonia (DYT-Tor1A), which is also inherited in an autosomal dominant manner, for example, only about 30 % of the mutation carriers develop the clinical phenotype [2], [3]. This relates to an *a priori* likelihood of only about 15–20 % that a first degree relative will actually become affected. However, genetic modifiers that can strongly influence penetrance should also be accounted for. For example, in DYT1 dystonia, a polymorphism on the non-mutated Tor1A allele (D216H) can reduce the penetrance of the disease-causing Tor1A three base pair deletion (c.907_909delGAG) to ∼ 3 % [4], [5]. Another example is GCH1-associated dopa-responsive dystonia (DRD, DYT/PARK-GCH1), in which reduced penetrance can also be observed, both in females and in males [6]. Male GCH1 mutation carriers, however, have a lower likelihood than females to develop DRD, and if they become affected they usually manifest the disease later in life [6]. Moreover, on average, they show a more parkinsonian phenotype, whereas women usually manifest with dystonia [6]. The mutation type may also have an influence on the penetrance of monogenic disorders. For example, the penetrance of triplications of the alpha synuclein gene is nearly 100 % to develop Parkinson’s disease whereas it is considerably lower in carriers of duplications [7]. Moreover, carriers of triplications develop Parkinson’s disease earlier in life, are more severely affected, and exhibit more rapid disease progression [8].

The type of mutation, genetic modifiers, gender and other factors may therefore have a significant impact on penetrance and expressivity. In the ideal of precision medicine, such knowledge should be incorporated into genetic counseling in the future to provide potential mutation carriers with a more comprehensive picture of the disease. More research, however, is required to generate more robust data on modifiers in usually very rare disorders.

## Rare diseases, databases and cohorts

Research in rare diseases including studies on penetrance and expressivity confronts scientists with the challenge that the essence of rare diseases is their low prevalence. Patient registries are an important instrument for addressing specific questions and making meaningful statements about these diseases. Different approaches to data collection can be used. At the level of individual working groups and research groups, highly specialized “patient collections”, e. g. of people with a specific mutation, are a feasible way to study, for example, the effect of a mutation on neurophysiological characteristics [9]. These studies are mostly based on case collections maintained by individual researchers. In the field of HD, the consolidation of many specialist outpatient clinics has led to the establishment of a special worldwide registry for HD with more than 70,000 registered clinical visits, which was, however, only possible with the financial support of a large foundation (https://enroll-hd.org).

Similar registries exist for other diseases, but all depend on funding from either foundations or the pharmaceutical industry. For example, the German Dystonia Registry, with initial funding from the BMBF, was able to establish a clinical registry with now more than 2,200 patients with dystonia (http://dystract.cio-marburg.de). EU resources built within the Rare disease actions financed in the last years lead to the creation of online resources as Eurobiobank and the Genome-Phenome analysis platform in the RD-connect project (https://platform.rd-connect.eu).

In Germany, the definition of special functions of the Centers for Rare Diseases by the Gemeinsamer Bundesausschuss (Federal Joint Committee) has now laid a structural foundation for the integrative and financial connection of patient registries to centers for rare disorders (Zentren für Seltene Erkrankungen (ZSE)).

The European Reference Networks (ERN) address the task to collect data across ERN centers. These centers commit themselves to making clinical data sets of patients with diseases they represent in the ERN available to the registry in a pseudonymized form. Here, the novel approach is to first get an overview of the prevalence of the diseases across centers and to be able to also identify patients in this meta-database for which only a reduced data set is available. With increasing harmonization of patient data in hospital information systems and platforms for querying specific data sets across sites, such as the CORD-MI initiative (https://www.medizininformatik-initiative.de/de/CORD), research and ultimately the treatment of rare diseases can be improved in the future. Making rare diseases visible in the health system through deeper coding of diseases with Orpha codes or Alpha IDs, even across sectors, would improve research and treatment of rare diseases in the future.

Such registries are an important starting point to identify affected mutation carriers but also to have a basis for studying unaffected family members to gain insights into the ‘real world’ penetrance of rare genetic diseases. Such a ‘recall-by-genotype’ approach has important ethical and legal implications which is highlighted in the next paragraph.

A special and very valuable subtype of data sets are cohorts. A cohort is a group of people with something in common, e. g. a disease that has been followed over a certain amount of time. There are several ways how the genetic and clinical examinations of cohorts aid the investigation of penetrance: 1) Genetic analyses of population-based cohorts inform researchers on the frequency of mutations in this population. 2) The same analyses identify mutation carriers. 3) Careful clinical examinations determine the status disease or control. Furthermore, new in-between stages such as “at risk”, “preclinical” and others have been defined and add to the question of disease/controls and thus penetrance. An example of a cohort study from us is EPIPARK. The EPIPARK cohort examines cases and controls annually and collects early and risk markers of PD. To study penetrance, follow-up at least until the age of the typical disease onset is crucial for evaluating the case/control status. This enables to address the question of the “in-between” stages such as at risk or preclinical. The study allows the investigation of mutation frequencies in cases and controls and rate of unaffected mutation carriers. As an example for an enriched cohort we briefly describe the TREND study. The TREND cohort has been recruited in a manner that enriched for several prodromal markers of PD, including olfactory loss, depression and possible REM-sleep behavior disorder. Regular 2-year follow-ups are being performed and several phenoconversions from control to PD have been observed during the 12 years since the inception of the study in 2009 highlighting the importance of follow-up in controls.

A recent collaborative, international approach used a survey to increase the number of patients with monogenic PD [10]. Information was collected from 8453 patients retrieved from 43 countries which is more than twice as much as reported in the literature. This approach enables identifying families with potential non-manifesting carriers to study reduced penetrance.

For dystonia on the other hand fewer studies are available, however two large registries the Dystonia Coalition and Dystract assembled the largest case numbers worldwide.

**Figure 1:** Neurodegeneration and the relationship to the development of symptoms indicating prodromal and manifest Parkinson’s disease. The loss of neurons in the substantia nigra is indicated in red. The increase in the severity of non-motor and motor symptoms is indicated in blue. Figure created with Biorender®.

1 – reduced sense of smell, 2 – gastrointestinal symptoms, e. g. constipation, 3 – sleep disorders, e. g. REM sleep behavior disorder, 4 – manifest Parkinson’s disease

## Ethical, legal and social implications

The research of reduced penetrance in healthy cohorts and clinical cohorts challenges some of our ethical and legal assumptions in the recruitment of patients. Generally, a potential study participant receives all the information necessary to make an autonomous decision about the participation during the enrolment phase of the study. In recall-by-genotype (RbG) studies, the unknowns make it difficult to streamline procedures for the consent and recontact and it must be assessed in every context (patients cohorts, healthy population cohorts etc.). In fact, the participants expectations may vary between different settings. With regard to the healthy cohorts described above, it is especially sensitive. While, within a patient cohorts, being recalled for deepen knowledge about some aspects is expected, it may be very different in healthy individuals.

As for in other recall by RbG studies, the recontact of individuals carrying a variant of unknown significance may reveal the reason for the recruitment to the study to the participant. However, at the time of recontact the inadvertent disclosure of sensitive genetic information may no longer be the wish of the individual. This knowledge about the genetic status may distress the study participant, result in concerns that relatives may carry the same variant, and could also affect individual reproductive decisions. The process of recruiting individuals carrying a variant thus requires further consideration, particularly the impact it has on the informed consent process, but also the potential need for genetic counselling as part of these studies.

As part of the PAREGEN consortium, empirical work was conducted to understand how best to design and conduct RbG studies within a strong co-produced ethical and legal framework [11].

Further work is required as in the context of RbG studies, but it is clear that careful consideration should be given to the informed consent process, with special attention to the type of information to be provided. Contact points dedicated to patients/participants in case of distress be provided to all study participants. An important example that highlights the importance of the framing of the disclosure of future disease has been shown for PD [12]. Here, PD patients retrospectively evaluated early risk disclosure before their disease onset with skepticism. However, if future disease status had been disclosed with options of early disease modification, such as potentially based on changes in diet or physical activity, agreement to the disclosure of future PD was markedly increased.

## Reduced penetrance or prodromal phase? Risk and prodromal markers in genetic forms of Parkinson’s disease

For sporadic, idiopathic PD the concept of a risk phase, a preclinical phase and a prodromal phase preceding the clinical diagnosis by many years or even decades has been widely established. The situation is different for dystonia which is usually not a degenerative disease and where no concept of a prodromal phase exists per to date. Prospective studies provided evidence on several risk markers indicating an increased PD risk, and several prodromal markers that may indicate early signs or symptoms of neurodegeneration (Fig. 1). Based on this (growing) body of evidence, the presence or absence of risk and prodromal markers in a healthy person can be used to calculate the probability of prodromal PD [13]. This approach proposed by the MDS research criteria for prodromal PD is currently still used for research purposes only. While this prediction approach for idiopathic PD will be continually refined as more evidence is gained, potential subtypes of prodromal idiopathic PD that might explain heterogeneity both in clinical as well as prodromal PD, are not yet incorporated [14]. However, for genetic forms of PD evidence of (non-)genetic risk markers and prodromal markers, and factors that may contribute to variable penetrance and expressivity, is still scarce. Possibly, several environmental factors (e. g. diet, sport, pesticide exposure), complex genetic factors, and their potential interplay with aging processes and/or PD pathogenesis, might not only contribute to the heterogeneity in (prodromal) idiopathic PD but also, and potentially differently, in non-idiopathic and monogenic PD. Moreover, complex polygenetic facets play an additional role for disease expressivity as suggested by findings of an association between higher polygenic risk scores (PRS) with an earlier age of (idiopathic) PD onset [15]. However, whether PRS are also linked to specific differences in prodromal features is still unclear.

So far, evidence suggests that the prodrome(s) of non-idiopathic/monogenic PD may differ from idiopathic in several aspects. Retrospective data of GBA-associated PD suggests a shorter and more severe prodrome regarding prodromal neuropsychiatric symptoms, autonomous dysfunction, sleep disturbances and olfactory dysfunction compared to idiopathic PD [16]. To which degree heterozygous carriers of recessive mutations (e. g. in PRKN, PINK1, DJ-1) are at an increased PD risk is still unclear and their prodromal characteristics and potential modifiers of disease expressivity requires further research.

Among genes involved in autosomal dominant monogenic PD, preclinical aspects in LRRK2 mutation carriers have been investigated the most. Non-manifesting LRRK2 G2019S mutation carriers have been shown to exhibit no difference in olfactory loss, sleep disturbances and most autonomous functions (except for slightly severe constipation and thermoregulatory function) compared to non-carriers [17]. While based on a smaller sample, R1441G variant carriers showed slightly higher depression and higher total autonomous dysfunction than non-carriers, and thus prodromal features may differ depending on specific variants within one gene. As even more rare, evidence of preclinical and prodromal features in SNCA and VPS35 mutation carriers with early onset familiar PD is scarce.

Risk factors, prodromal features and modifiers may differ depending on the specific gene or even specific variants and associated pathomechanisms involved, e. g. mitochondrial, lysosomal, autophagy, α-synuclein dysfunction. Therefore, a differentiating investigation of specific risk factors and prodromal markers as well as potential modifiers of disease penetrance and expressivity is needed to characterize and disentangle the prodromes of idiopathic, non-idiopathic and fully monogenic PD. Given the relatively small samples of individual cohorts and required longitudinal data (of non-manifesting carriers), collaboration among researchers is pivotal to advance the understanding of prediagnostic and prognostic features in genetic forms of PD and early intervention strategies.

Importantly, modifiers of disease penetrance or expressivity including age at onset may also indicate novel targets of early prevention and intervention strategies. Ideally, such early intervention should be feasible on a population level, with or without knowledge of genetic status of an individual subject. Moreover, such intervention strategies should carry little side-effects and should be actionable, such as life-style (diet, physical activity) intervention programs or drugs with modifying effects that can be repurposed.

## Conclusions and perspectives

Reduced penetrance is an important but underreported phenomenon in monogenic movement disorders. These disorders, however, are often exceedingly rare and penetrance estimation is usually based on single families. Therefore, large-scale mutation analyses in different cohorts of patients with common movement disorders and controls are necessary for an unbiased approach to identify manifesting and non-manifesting carriers of disease-causing mutations. Cross-sectional analyses, however, harbor the risk that unaffected mutation carriers could develop the disease later in life highlighting the necessity to longitudinally follow-up mutation carriers to get a better estimate on phenoconversion rates. Moreover, deep phenotyping of unaffected mutation carriers is essential to identify prodromal or risk markers that may already be present at the time of study inclusion. This is of particular relevance for neurodegenerative movement disorders, e. g. monogenic PD. The identification of patients in the prodromal phase of a neurodegenerative disorder provides an intriguing opportunity to initiate disease-modifying treatments earlier in the disease course. Important challenges remain as the prediction who will convert from a healthy individual to a clinical patient and in whom penetrance remains completely reduced is still associated with great uncertainty. Longitudinal follow-up deep phenotyping of non-manifesting mutation carriers identified from population-based control cohorts will provide important insights on the interplay of reduced penetrance, clinical expressivity and prodromal disease stage. Although several initiatives exist and an important infrastructure with centers of rare diseases arise, the development of an overarching nation-wide database connecting different centers with standardized protocols and secured funding for a long-term approach, e. g. through government agencies, are pre-requisites to fulfill this goal.
